# Application of electromagnetic navigation guidance technology in enteral nutrition support for a case of severe acute pancreatitis with duodenal fistula: a case report

**DOI:** 10.3389/fmed.2025.1615071

**Published:** 2025-11-11

**Authors:** Jun Zhou, Yalan Huang, Yuping Rong, Xiusi Wang, Qiang Yang

**Affiliations:** 1 Department of Pancreatic Surgery, Renmin Hospital of Wuhan University, Wuhan, China; 2 Department of Hepatobiliary and Pancreatic Oncology, Renmin Hospital of Wuhan University, Wuhan, China

**Keywords:** severe acute pancreatitis, duodenal fistula, electromagnetic navigation guidance, enteral nutrition, nasojejunal feeding tube

## Abstract

A female patient with severe acute pancreatitis (SAP) complicated by infected pancreatic necrosis (IPN) developed a descending duodenal fistula during the sixth week of disease progression. Enteral nutrition (EN) delivery was hindered by occlusion of the nasojejunal tube in another hospital. We successfully placed a nasojejunal tube under electromagnetic navigation and initiated enteral nutrition support. This report highlights the application of this nutritional support technology in patients with high-output complex intestinal fistula.

## Introduction

Severe acute pancreatitis (SAP) is a common critical gastrointestinal disorder, with the adverse clinical outcomes in most cases. SAP is characterized by a systemic inflammatory response-induced hypermetabolic state and accelerated protein catabolism. Patients with SAP should be considered at high nutritional risk due to the hypercatabolic state and the critical impact of nutritional status on clinical outcomes (1). Therefore, adequate nutritional therapy constitutes a therapeutic cornerstone in the management of SAP. Numerous studies have demonstrated that enteral nutrition (EN) is safer and better tolerated than parenteral nutrition (PN) in SAP with markedly reduced rates of complications, multiple organ failure (MOF), and mortality (2). Consequently, EN is considered an essential component of nutritional therapy for SAP patients.

Gastrointestinal fistulae represent a severe complication of SAP (3). High-position gastrointestinal fistulae often lead to significant digestive fluid loss and render oral intake or nasogastric nutritional therapy infeasible, making jejunal feeding the optimal therapeutic choice. Current jejunal tube placement methods (blind insertion, ultrasound/X-ray guidance, endoscopic placement, or jejunostomy) face limitations in SAP patients with duodenal fistulae due to procedural complexity, high complication rates, or invasiveness (4).

Electromagnetic navigation technology is a positioning technique based on electromagnetic sensing that determines target location by measuring electromagnetic wave propagation time and signal strength. Initial exploratory applications of magnetic guidance for adjunctive feeding tube placement were documented as early as 2000 (5). By 2008, dedicated electromagnetic navigation systems had been introduced into clinical practice. Compared to conventional techniques, the electromagnetic-guided approach offers reduced invasiveness, eliminates deep sedation requirements, and enables placement by trained allied health professionals without direct physician involvement. This technique is currently recommended as a novel nasoenteric tube placement method due to its capacity to minimize feeding delays and eliminate radiographic localization requirements in critically ill patients, thereby achieving significant cost savings (6, 7). While prior studies applied Electromagnetic navigation technology in uncomplicated SAP (8), its use in proximal intestinal fistulae remains unreported.

The anatomical disruption in fistulae creates unique challenges. Therefore, we report a case of SAP with descending duodenal fistula in which electromagnetic navigation technology enabled safe and convenient bedside placement of a nasojejunal feeding tube for enteral nutrition therapy.

## Case report

**Figure 1:**
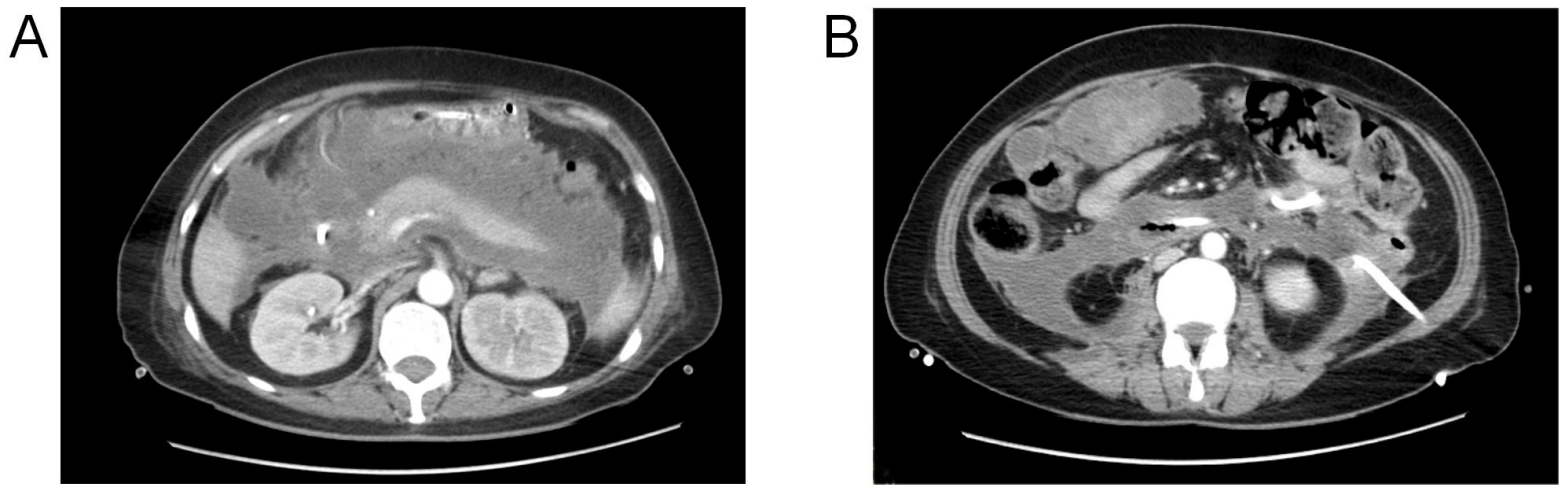
**(A**) Peri-pancreatic necrosis demonstrated on contrast-enhanced computed tomography (CECT); **(B)** retroperitoneal drainage catheter (12Fr).

A 49-year-old female patient presented with epigastric pain and was diagnosed with severe acute pancreatitis (SAP) at an external hospital. Although early treatment improved her organ function, she developed fever at 4 weeks of disease progression. Computed tomography (CT) revealed extensive peripancreatic and retroperitoneal necrosis (infected pancreatic necrosis, IPN), classified as Balthazar grade III (Figure 1A) (9). Bilateral retroperitoneal catheter drainage (12Fr pigtail catheters) and aggressive anti-infective therapy led to significant fever reduction (Figure 1B). Unfortunately, at 6 weeks of disease progression, copious yellowish-brown digestive fluid was observed from the right abdominal drainage tube, strongly suggesting an upper gastrointestinal fistula. Concurrently, enteral nutrition was hindered by occlusion of the nasojejunal feeding tube. The patient was transferred to our hospital at 6 weeks of disease progression. Physical examination: Height 164 cm, weight 45 kg (BMI 16.7 kg/m^2^), temperature 36.7 °C, BP 128/63 mmHg, pulse 113 bpm, respiratory rate 22/min. Nutritional assessment via Nutrition Risk Screening 2002 (NRS-2002) scored 5 (malnutrition risk), with Global Leadership Initiative on Malnutrition (GLIM) criteria confirming severe malnutrition (10). Laboratory findings: albumin 30.2 g/L, prealbumin 0.129 g/L, CRP 215.8 mg/L, hemoglobin 78 g/L, potassium 3.12 mmol/L, phosphorus 0.49 mmol/L, sodium 135.5 mmol/L, and calcium 1.82 mmol/L.

**Figure 2:**
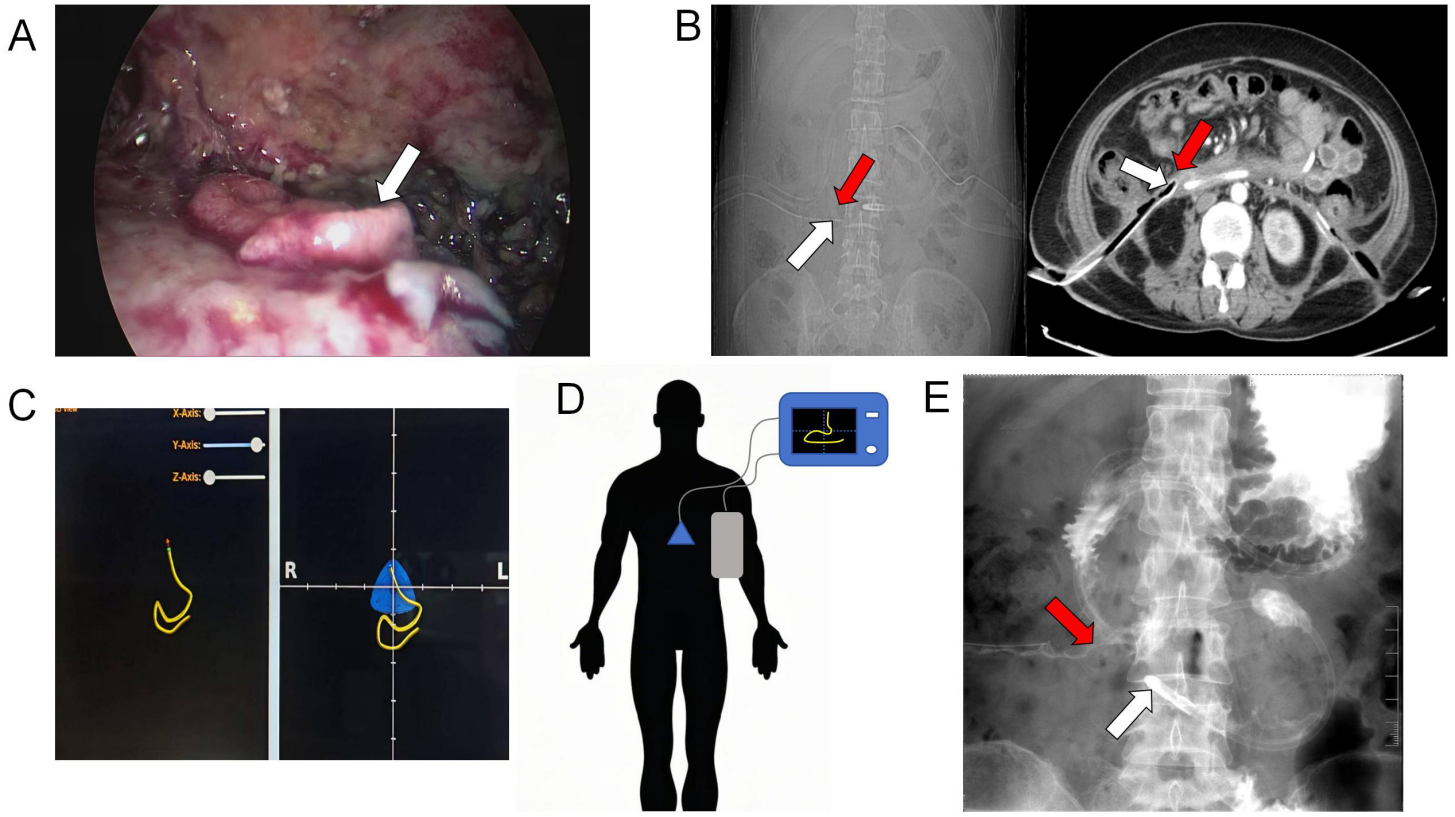
**(A)** Intraoperative visualization of the duodenal fistula orifice (white arrow); **(B)** Post-operative CT demonstrated the position of the drainage tube tip (white arrow) and the location of the duodenal fistula (red arrow); **(C, D)** use of electromagnetic navigation for inserting the nasojejunal tube; **(E)** the jejunal feeding tube tip position (white arrow) and duodenal fistula orifice localization (red arrow) was achieved via water-soluble iodinated contrast radiography.

Video-assisted retroperitoneal debridement confirmed a perforation in the anterior wall of the descending duodenum via methylene blue leakage test (Figure 2A). We removed most necrotic tissue and placed a 32Fr drainage catheter adjacent to the fistula. Post-operative drainage was effective, with the fistula orifice location confirmed by assessing the spatial relationship between the drainage catheter tip and the duodenum on post-operative CT images (Figure 2B). However, severe malnutrition necessitated urgent enteral access. Nutritional intervention: Due to severe malnutrition and risks of conventional methods (blind insertion risks tube misplacement into necrotic cavities; ultrasound limitations in duodenal imaging; endoscopic air insufflation risks fistula expansion), In alignment with the patient's and family's preference for a non-surgical approach, bedside electromagnetic navigation guided nasojejunal tube placement was performed. Utilizing real-time electromagnetic tracking and 3D spatial mapping, the catheter tip was successfully positioned within the proximal jejunum (Figures 2C, D). Post-procedural water-soluble iodinated contrast radiography confirmed proper positioning of the catheter tip distal to the ligament of Treitz (Figure 2E).

**Figure 3:**
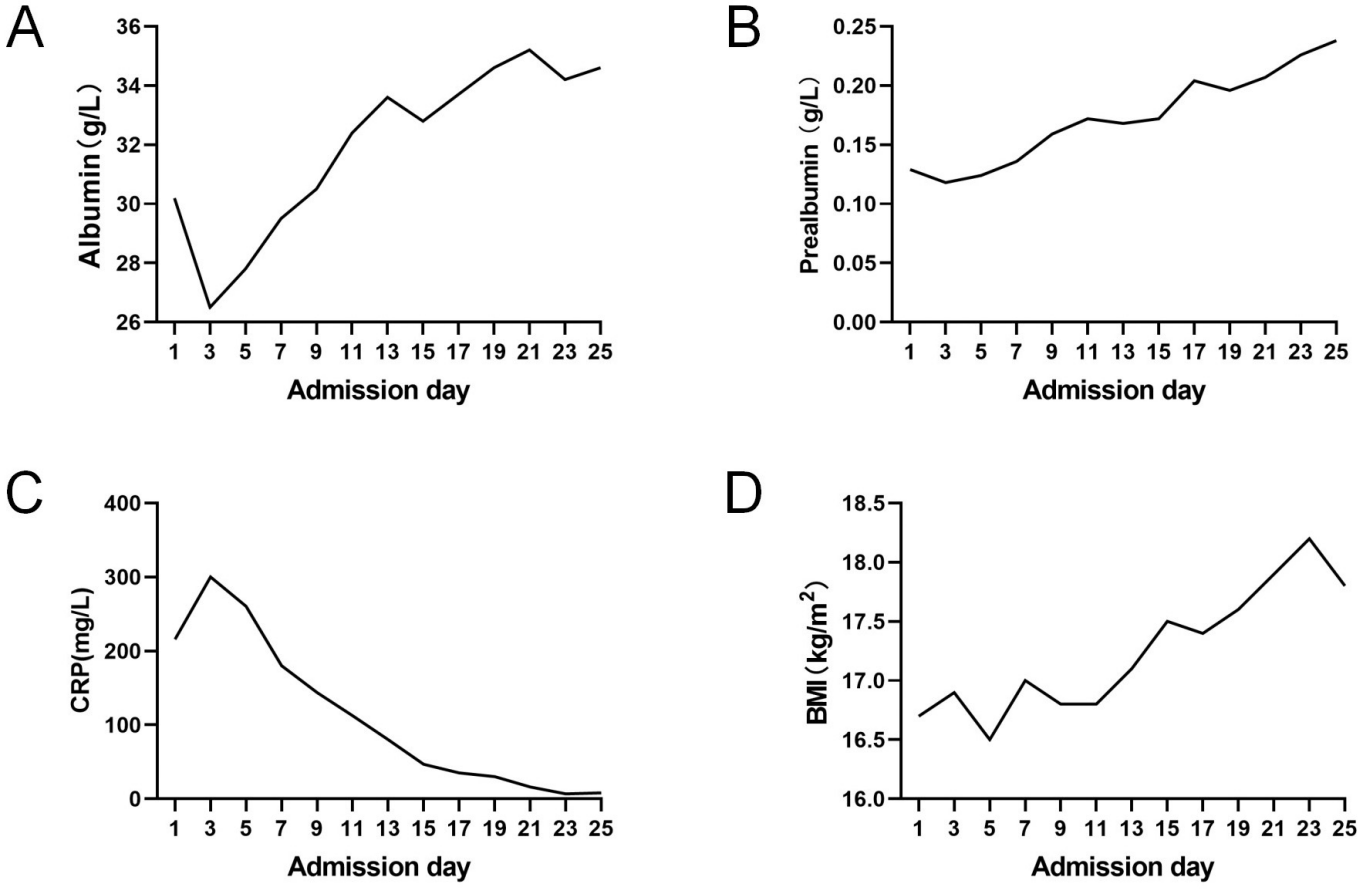
**(A–D)** The trends of laboratory examinations results.

A protocolized nutritional regimen was implemented for this patient, aligned with the 2024 ESPEN (European Society for Clinical Nutrition and Metabolism) guidelines for acute pancreatitis (11). The therapeutic targets were set at protein 1.2–2.0 g/kg/day and calories 20–25 kcal/kg/day. No enteral formula reflux, abdominal distention, diarrhea, or refeeding syndrome occurred. Post-treatment laboratory trends demonstrated gradual improvement (Figure 3). The surgical drain was removed at 4 weeks post-procedure following confirmation of fistula closure. The patient successfully transitioned to oral alimentation with gradual EN weaning, maintaining >75% of target caloric intake via oral route by discharge.

## Discussion

Enteric fistulae represent a severe late-stage complication of SAP, with an incidence rate ranging from 8 to 19% (12). Although uncommon, their development portends detrimental clinical outcomes (13). Comparative analyses reveal that SAP patients with duodenal fistulae exhibit significantly higher rates of multiple organ failure (MOF), gastrointestinal hemorrhage, percutaneous catheter drainage interventions, surgical interventions, prolonged hospitalization, and elevated hospitalization costs relative to uncomplicated SAP patients (14). The disruption of intestinal continuity compromises nutritional supplementation, while concurrent infections and other factors induce a hypercatabolic state, resulting in elevated nutritional risk in SAP patients complicated by enteric fistulae (15).

Consequently, aggressive nutritional support is imperative. The cornerstone principle of nutritional intervention mandates prioritizing EN in patients with functional and safely utilizable gastrointestinal tracts (16–18). However, SAP patients with proximal enteric fistulae present unique clinical challenges, particularly regarding the establishment of safe nutritional access pathways (19). Current clinical strategies for jejunal feeding tube placement include blind insertion, ultrasonographic guidance, fluoroscopic assistance, endoscopic placement, and surgical jejunostomy (20). In SAP patients with proximal fistulae, blind insertion carries significant risks, as catheters may extrude through the fistula orifice rather than remaining within the intestinal lumen. Ultrasonographic guidance is constrained by suboptimal imaging visualization due to tissue edema, intraluminal gas interference, and unfavorable body habitus. Fluoroscopic techniques require specialized equipment and operator expertise, limiting bedside implementation while exposing patients to radiation risks. Endoscopic nasojejunal tube placement necessitates intestinal lumen insufflation, potentially exacerbating fistula enlargement.

In this case, following a comprehensive assessment by the multidisciplinary nutrition support team, a non-conventional approach was selected for nasojejunal tube placement due to the patient's unique clinical status. After intravenous administration of 10 mg metoclopramide to enhance gastric and duodenal motility, the nasoduodenal tube was advanced under imaging guidance. Fluoroscopic imaging confirmed successful passage of the catheter through the gastric lumen along the greater curvature and subsequent trans-pyloric progression. Catheter tip rigidity was dynamically modulated by manipulating guidewire configuration and positioning, enabling precise navigation to the duodenal horizontal segment. Final fluoroscopic verification in triaxial planes (X, Y, and Z) confirmed optimal placement of the catheter tip within the proximal jejunum. The procedure was completed without procedural complications or patient-reported adverse symptoms.

Electromagnetic navigation guidance technology utilizes the spatial distribution profile of quasi-static magnetic fields to enable real-time measurement of instrument spatial coordinates and angular orientation (21). This technology has gained increasing clinical adoption in medical applications, with jejunal feeding tube placement emerging as a prominent implementation (22, 23). Conventionally, duodenal fistulae are considered a contraindication for non-visualized jejunal feeding tube placement. However, duodenal fistulae were safely managed in this case through abdominal CT-confirmed duodenal morphology and fistula localization, combined with real-time electromagnetic navigation tracking and dynamic modulation of catheter tip rigidity via guidewire configuration manipulation and positional adjustments, thereby ensuring continuous intraluminal positioning without peritoneal entry. Post-placement verification was achieved by injecting methylene blue saline solution through the nasoenteric tube, with absence of blue effluent in the retroperitoneal drainage tube reconfirming correct tip positioning.

This technique overcomes the critical limitation of conventional methods in fistula settings. Electromagnetic navigation guided catheterization demonstrates significant advantages over conventional methods:

- Precision: real-time electromagnetic tracking enables direct jejunal catheter placement while circumventing the duodenal fistula orifice and necrotic cavities, a critical capability for patients with altered anatomical architecture.
- Safety: eliminates risks of fistula enlargement secondary to endoscopic insufflation and avoids fluoroscopic radiation exposure, aligning with radiation safety protocols.
- Convenience: bedside implementation within intensive care units is feasible, with capability for repeatable procedural attempts without requiring patient transfer.
- Feasibility: successful catheterization was achieved despite concurrent severe malnutrition and retroperitoneal inflammatory infiltration, demonstrating technical applicability in high-risk patient cohorts.

## Conclusion

This case demonstrates the successful application of electromagnetic navigation guidance technology for bedside nasojejunal tube placement in a high-risk SAP patient with duodenal fistula, where conventional methods were contraindicated. Electromagnetic navigation guidance enabled precise enteral access establishment while circumventing anatomical disruptions, facilitating uncomplicated enteral nutrition support. This approach represents a promising alternative for proximal intestinal fistulae management in critical settings. Future studies should validate its efficacy across diverse fistula types and clinical scenarios.

## Data Availability

The raw data supporting the conclusions of this article will be made available by the authors, without undue reservation.
